# Using Bayesian state-space models to understand the population dynamics of the dominant malaria vector, *Anopheles funestus* in rural Tanzania

**DOI:** 10.1186/s12936-022-04189-4

**Published:** 2022-06-03

**Authors:** Halfan S. Ngowo, Fredros O. Okumu, Emmanuel E. Hape, Issa H. Mshani, Heather M. Ferguson, Jason Matthiopoulos

**Affiliations:** 1 Department of Environmental Health & Ecological Sciences, Ifakara Health Institute, Ifakara, Tanzania; 2 Institute of Biodiversity, Animal Health and Comparative Medicine, University of Glasgow, Glasgow, UK; 3 School of Public Health, University of the Witwatersrand, Braamfontein, Republic of South Africa; 4 School of Life Science and Bioengineering, Nelson Mandela African Institution of Science & Technology, Arusha, Tanzania

**Keywords:** Anopheles *funestus*, State space model, Population dynamic, Seasonality, Abundance, Density dependence

## Abstract

**Background:**

It is often assumed that the population dynamics of the malaria vector *Anopheles funestus*, its role in malaria transmission and the way it responds to interventions are similar to the more elaborately characterized *Anopheles gambiae*. However, *An. funestus* has several unique ecological features that could generate distinct transmission dynamics and responsiveness to interventions. The objectives of this work were to develop a model which will: (1) reconstruct the population dynamics, survival, and fecundity of wild *An. funestus* populations in southern Tanzania, (2) quantify impacts of density dependence on the dynamics, and (3) assess seasonal fluctuations in *An. funestus* demography. Through quantifying the population dynamics of *An. funestus*, this model will enable analysis of how their stability and response to interventions may differ from that of *An. gambiae *sensu lato.

**Methods:**

A Bayesian State Space Model (SSM) based on mosquito life history was fit to time series data on the abundance of female *An. funestus *sensu stricto collected over 2 years in southern Tanzania. Prior values of fitness and demography were incorporated from empirical data on larval development, adult survival and fecundity from laboratory-reared first generation progeny of wild caught *An. funestus*. The model was structured to allow larval and adult fitness traits to vary seasonally in response to environmental covariates (i.e. temperature and rainfall), and for density dependency in larvae. The effects of density dependence and seasonality were measured through counterfactual examination of model fit with or without these covariates.

**Results:**

The model accurately reconstructed the seasonal population dynamics of *An. funestus* and generated biologically-plausible values of their survival larval, development and fecundity in the wild. This model suggests that *An. funestus* survival and fecundity annual pattern was highly variable across the year, but did not show consistent seasonal trends either rainfall or temperature*.* While the model fit was somewhat improved by inclusion of density dependence, this was a relatively minor effect and suggests that this process is not as important for *An. funestus* as it is for *An. gambiae* populations.

**Conclusion:**

The model's ability to accurately reconstruct the dynamics and demography of *An. funestus* could potentially be useful in simulating the response of these populations to vector control techniques deployed separately or in combination. The observed and simulated dynamics also suggests that *An. funestus* could be playing a role in year-round malaria transmission, with any apparent seasonality attributed to other vector species.

**Supplementary Information:**

The online version contains supplementary material available at 10.1186/s12936-022-04189-4.

## Background

*Anopheles funestus* is one of the major malaria vectors in Africa and is widely distributed across the continent [1, 2]. With the exception of *Anopheles gambiae *sensu stricto (*s.s*.), the species appears to have higher vectorial capacity than many other members of the *Anopheles gambiae* complex [3–8]. *Anopheles funestus* makes a higher contribution to transmission than *An. gambiae *sensu lato (*s.l*.) in numerous parts in sub-Saharan Africa [6, 9–12]; particularly in settings where *An. gambiae* abundance has plummeted due to either effective indoor-based vector control interventions [13, 14] or environmental change. It is hypothesized that *An. funestus* persistence despite the recent scale-up of insecticide–treated nets may have been facilitated by their earlier development of strong physiological resistance [15].

*Anopheles funestus* is typically grouped with *An. gambiae s.l.* when modelling transmission and formulating policies for malaria vector control [16–18]. The lack of explicit consideration of *An. funestus* ecology and transmission potential may be partially due to this species having been relatively neglected compared to *An. gambiae*. Comparatively the ecology of *An. funestus s.l*. is less well understood, and it is much more difficult to maintain under insectary or semi-field conditions [19]. However, this species has several unique ecological features, such as its different larval habitat and dry season persistence [20], that could give rise to distinct population dynamics and differentiate its response to core and supplementary interventions. For example, *An. funestus* prefers larger aquatic habitats that are semi-permanent or permanent throughout the year, and contain clear water with some emergent vegetation [20]. This differs from *An. gambiae s.s*. which generally prefer small temporary habitats, such as puddles, ditches or animal hoof prints [20, 21], or *Anopheles arabiensis*, which can breed extensively in rice fields and other sunlit open pools [1]. The use of more permanent larval habitats means that *An. funestus* has greater persistence through the driest periods of the year compared to *An. gambiae* [22], whose habitats evaporate quickly in the absence of rainfall [21, 23]. This ecological feature means that the seasonal phenology of *An. funestus* and its response to aquatic microclimate differs from *An. gambiae* [21, 22, 24]; and could thus generate differential response to seasonally-targeted interventions, such as Indoor Residual Spraying (IRS) and larviciding.

Differential use of aquatic habitats may also impact the relative importance of key intrinsic drivers of mosquito population dynamics such as density dependence. Density dependence in malaria vectors occurs during larval development as a product of competition for space and nutritional resources [25, 26]. In space-limited habitats, high larval densities can influence larval development rates and survival, but also subsequent adult fitness traits such as body size, survival, fecundity and mating success [27–30]. While there is evidence that density dependence is an important driver of *An. gambiae* population dynamics [25], the relative importance of this process for *An. funestus* is unknown. Given that larval crowding and competition are less likely within the larger habitats preferred by *An. funestus,* density dependence is hypothesised to may be less pronounced for this vector species. Quantifying the strength of density dependence is important to inform the ease with which vector populations can be suppressed and how quickly they can recover [26, 27, 31].

Models of vector population dynamics and their response to interventions must be parameterised by reliable estimates of their demography and fitness. For vectors in the *An. gambiae* complex, such estimates are often acquired from insectary and semi-field studies [32–37] as well as field studies. Similar data has been difficult to obtain for *An. funestus* because of its poorly understood ecology and the difficulties of creating laboratory colonies; which so far has been achieved on only two occasions [11, 19, 38]. State-space models (SSM) provide an alternative approach to indirectly estimate these parameters by fitting a population dynamics model to observed time series data [39, 40]. These models are widely used in other fields of ecology and conservation biology to investigate the population dynamics of other animals [39, 41] and guide management decisions [41]. However, these models have so far had limited update in medical entomology. Given data on population fluctuations are available, these models can infer and estimate plausible demographic rates that could generate the observed dynamics [42].

SSMs are time-series models that distinguish between two stochastic components, namely, process (i.e. biological), which captures sequential dependencies between population components (e.g. eggs, larvae, pupae) and an observation component, which captures and corrects for biases and imprecisions in the data-collection process. Prior knowledge of the model parameters is used to bolster the information content of the time series data with existing expert or laboratory data and uncertainty in estimates. Population projections are then quantified on the basis of posterior probability distributions for parameters and population states. SSMs have recently been used to elucidate the dynamics and impacts of interventions on malaria vectors in laboratory and semi field populations [32, 33], but have not yet been applied to estimate *An. funestus* vector demographics in the wild. Here, an innovative SSM application was developed to describe the dynamics of wild *An. funestus* populations in Tanzania, and use it to assess extrinsic (environmental) and intrinsic (density dependence) drivers of their fitness and demography for the first time. Empirical data from laboratory experiments on *An. funestus* colonization [19] were incorporated together with the wild population data to develop an SSM. Time series field data collected in 2015 [6] and 2018 south-eastern Tanzania, and corresponding environmental information were used to validate the model. Specific aims were: (1) to accurately reconstruct the population dynamics, survival and fecundity of wild *An. funestus* populations in southern Tanzania, (2) quantify the effects of density dependence on the dynamics, and (3) to identify and quantify seasonal variations in *An. funestus* demography.

## Methods

### Time series data on wild *An. funestus* populations

**Fig. 1:**
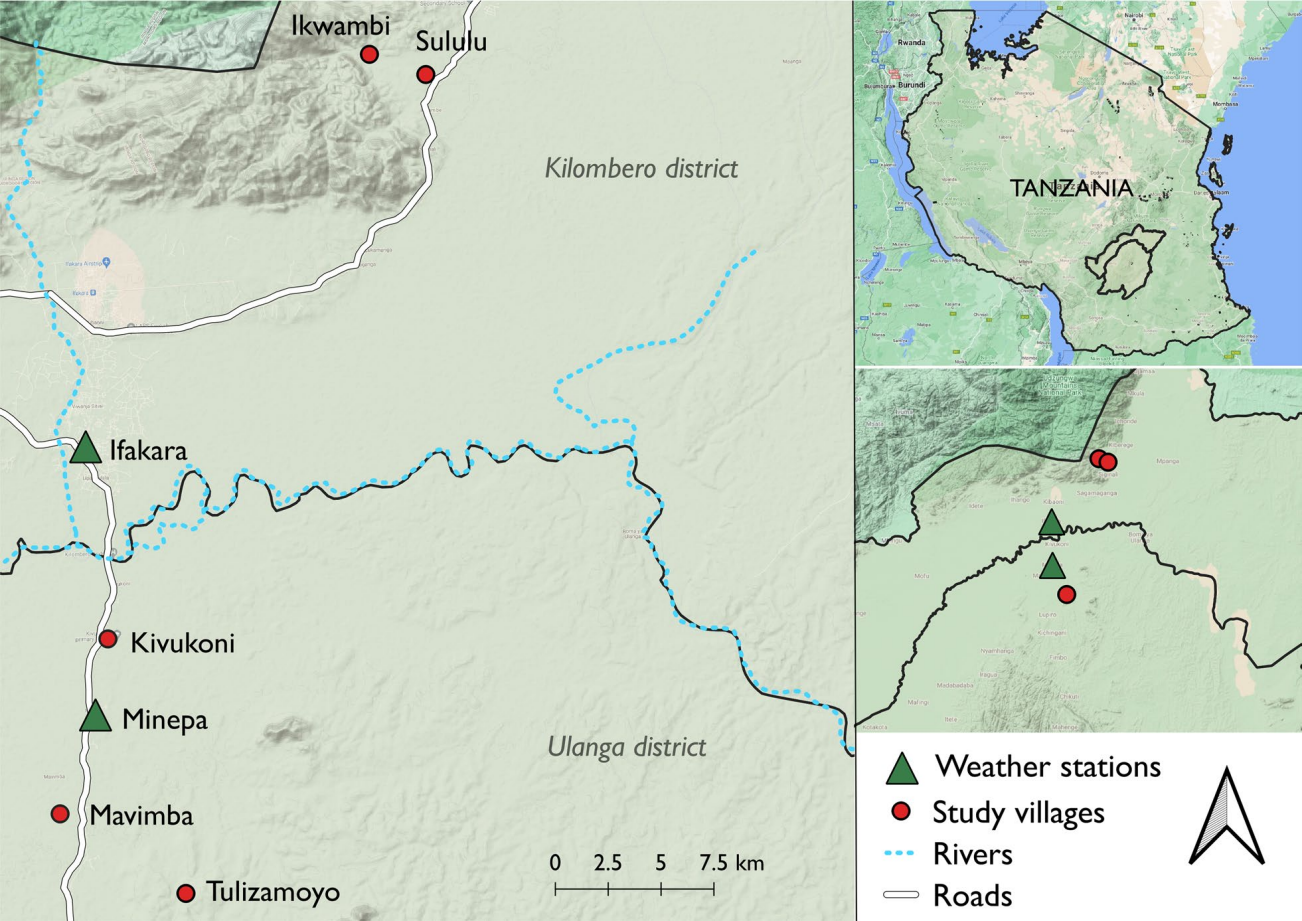
A map depicting the locations of various study villages where mosquito sampling was carried out in 2015–2016 and 2018–2019 (*Kindly prepared by Najat Kahamba*)

Indoor densities of female *An. funestus s.l.* adults were recorded over 12 months of entomological surveys conducted in three villages (Tulizamoyo, Ikwambi and Sululu) in Kilombero (8.1539ºS, 36.6870ºE) and Ulanga (8.3124ºS, 36.6879ºE) districts, south-eastern Tanzania from June 2018 to May 2019 (Fig. 1). The villages were selected because of the high abundance of *An. funestus s.l.* within which *An. funestus s.s.* is the dominant sibling species (93%) [1]. Annual rainfall was 1200–1800 mm, and temperature, 20–32 °C. CDC Light traps [43] were used to sample host-seeking mosquitoes from 6 pm to 6am for 5 days per week, 4 weeks a month for 12 months in 10–15 houses per village. The houses were randomly selected and consent obtained from household heads, mosquito sampling were done repeatedly in these houses. The mosquitoes were sorted by taxa and sex, and females further classified as unfed, blood-fed or gravid. Daily climatic data (rainfall and temperature) were obtained from a weather station, approximately ~ 20 km from the farthest village.

To complement this, additional data on *An. funestus* were extracted from a 2015 dataset from three other villages in Ulanga district (Mavimba, Minepa and Kivukoni) (Fig. 1) [6]. These data were collected five days per week for a period of 12 months. This data allowed us to fit the model simultaneously to multiple time series so that it could learn hierarchically from *An. funestus* trajectories enfolding in different years and locations. This additional data has previously been described elsewhere and used to demonstrate the epidemiological dominance of *An. funestus*, which now contributes > 85% of all malaria infections in the region [6].

### Prior information on life-history and gonotrophic cycle stages

**Fig. 2:**
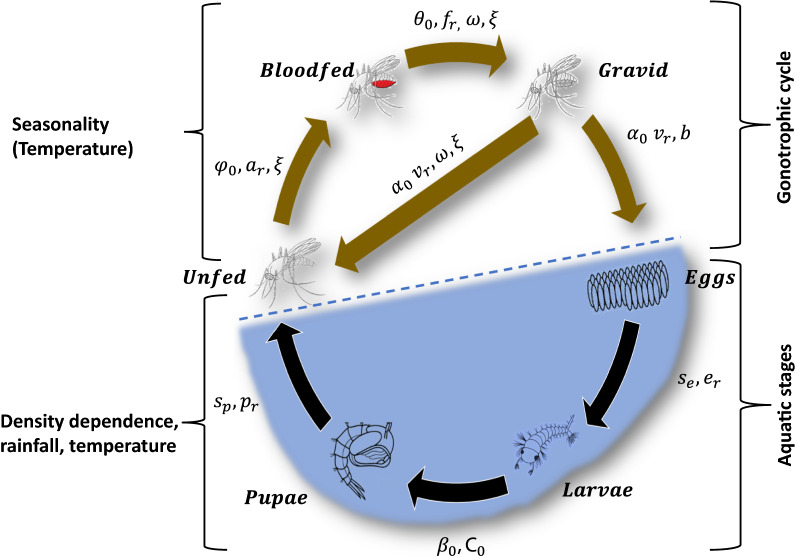
Schematic representation of the state-space population model showing different life stages compartment (circles) and flows (arrows) of *Anopheles funestus*. Abundance data were only available for unfed, blood-fed and gravid stages. The model assumes that once a gravid mosquito has laid eggs, they return to the unfed stage. The annotations are described in Table 1. The model incorporates six life stages (eggs, larvae, pupae, unfed, bloodfed and gravid) of *An. funestus*

Female *An. funestus* adults collected from the same three villages in 2018 were maintained in insectary conditions for one generation to estimate baseline fitness traits as already described in Ngowo et al. [19] and in Fig. 2. Data collected from this 1^st^-generation laboratory progeny included: (a) proportions of eggs that hatched into larvae, larvae that transitioned to pupae, and of pupae that emerged into adults, (b) the length of the transition periods (days) between life stages [(i) eggs to 1st instar larvae, (ii) 1st instar larvae to pupae, and (iii) pupae to unfed adult female (1 day post emergence)], (c) transition period of adult females between three different stages of their gonotrophic cycle, i.e. unfed, blood-fed and gravid.

The gonotrophic cycle starts with ‘unfed’ females who transition to ‘blood-fed’ after obtaining a blood meal. In the wild, the first gonotrophic cycle usually starts after unfed females have mated [44]; which is assumed to happen soon after emergence. In insectary experiments, females had access to males immediately on emergence. As the blood meal is digested, blood-fed females transition into the ‘gravid’ state during which eggs develop. Gravid females then oviposit their eggs into aquatic habitats and return to the ‘unfed’ stage with the cycle begins again (Fig. 2). In the wild, the rate of transition between these gonotrophic stages is governed by both intrinsic and extrinsic environmental conditions including the availability of blood-meals and oviposition sites [45]. In the insectary, the first blood-meal (arm-feeding) was offered 5 days post emergence to ensure individuals had sufficient time for mating.

Per capita fecundity was defined as the number of eggs laid per fully bloodfed adult female. The proportions surviving between life-history stages or gonotrophic stages were calculated as the inverse of the number of days required to transit from one stage to the next:

1 $${s}_{m}={({j}_{m})}^{1/r}$$

Here, $m$ is the life cycle stage, $j$ is proportion survived as the percentage of the total from preceding life stage, $r$ is the average number of days it took to transit from one life stage to another, and $s$ is the daily survival within the stage (Table 1).

**Table 1:** Priors as used in the state-space population models of *Anopheles funestus* and the estimated posteriors mean and 95% credible intervals

Parameter		Prior distribution				Posterior distribution	
Notation	Description	Type	Source	Mean	95-percentiles	Mean	95-percentiles
${s}_{e}$	Eggs daily survival rate	Beta	This study	0.794	[0.619, 1]	0.789	[0.776, 0.804]
$\lambda$	Eggs development period	Beta	This study	0.5	[0.4, 0.6]	0.499	[0.485, 0.514]
${\beta }_{0}$	Baseline larval daily survival	Beta	This study	0.923	[0.801, 1]	0.950	[0.943, 0.956]
${C}_{0}$	Baseline larval development period	Beta	This study	0.063	[0.055, 0.071]	0.063	[0.062, 0.064]
${s}_{p}$	Pupae daily survival rate	Beta	This study	0.941	[0.874, 1]	0.944	[0.930, 0.950]
${p}_{r}$	Pupae development period	Beta	This study	0.522	[0.253, 0.792]	0.525	[0.506, 0.546]
${\varphi }_{0}$	Baseline unfed daily survival	Beta	This study	0.935	[0.877, 0.992]	0.937	[0.933, 0.941]
${a}_{r}$	Unfed development period	Beta	This study	0.20	[0.19, 0.21]	0.200	[0.198, 0.201]
${\theta }_{0}$	Baseline blood-fed daily survival	Beta	This study	0.807	[0.654, 0.961]	0.810	[0.799, 0.820]
${f}_{r}$	Blood-fed daily transition rate	Beta	This study	0.25	[0.05, 045]	0.269	[0.256, 0.280]
${\alpha }_{0}$	Baseline gravid daily survival	Beta	This study	0.904	[0.848, 0.961]	0.903	[0.899, 0.907]
${v}_{r}$	Gravid daily transition rate	Beta	This study	0.333	[0.133, 0.533]	0.311	[0.297, 0.324]
${b}_{0}$	No. eggs/female (Per capita fecundity)	Beta	This study	80	[60, 100]	78	[74, 80]
$\xi$	Coefficient of variability	Beta	Uninformative prior	0.5	[0.1, 0.9]	0.79	[0.810, 0.825]
${\omega }_{\eta =f}$	Coefficient of “Trap biasness” for the blood-fed	Beta	Msugupakyula et al. [58] and Kaindoa et al. [6]	0.1	[0.05, 0.15]	0.122	[0.117, 0.127]
${\omega }_{\eta =v}$	Coefficient of “Trap biasness” for the gravid			0.505*${\omega }_{f}$	[0.025,0.076]	0.062	[0.059, 0.064]

Refers to an interaction between 1-week cumulative rainfall and density dependency

### Biological process components of the Bayesian SSMs

#### Daily survival

The daily survival of larval stages was assumed to be the same for all instar stages. Adult survival was assumed to be the same for unfed, blood-fed and gravid females. Survival probabilities (${s}_{l}, {s}_{a}, {s}_{f}, {s}_{v}$) were linked to their covariates through a *logit* transformation of linear predictors (here, subscripts $l, a, f, v$ refer to larvae, unfed, blood-fed and gravid, respectively). Pupal (${S}_{p}$) and egg (${S}_{e}$) survival probabilities were considered to be independent of any climatic and density-dependent covariates, and were treated as a binomial distribution, with baseline rates assigned priors as described in Eq. 16–17.

A range of covariates hypothesized to be associated with the demography of *An. funestus* were incorporated to allow baseline larval and adult survival to vary with environmental conditions. Rainfall (current and 1 week-lagged) and temperature were incorporated into the larval survival model. Rainfall regulates the availability and permanence of aquatic habitats, thus influencing both survival and carrying capacity of larval habitats [46]. Density dependence was incorporated into the model of larval survival [25] to assess whether this could improve the fit of the adult population dynamics model. Additionally, the speed of larval development was modelled as a function of temperature based on its known importance [47, 48]. The daily survival of larvae was thus defined as a function of daily rainfall (current and lagged), daily temperature and density dependence. The daily survival rates (lowercase ${s}_{l}\left(t\right)$) of larvae were estimated through a logit transformation of linear predictors (uppercase ${S}_{l}\left(t\right)$).

2 $${s}_{l}\left(t\right) =\frac{\mathrm{exp}\left({S}_{l}\left(t\right)\right)}{1+\mathrm{exp}\left({S}_{l}\left(t\right)\right)}$$

Specifically, ${S}_{l}\left(t\right)$ is written as a function of both intrinsic and extrinsic drivers:

3 $${S}_{l}\left(t\right) = {\beta }_{0}-{\beta }_{1}{R}_{\left(t-1\right)}-{\beta }_{2}{D}_{\left(t-1\right)}\left(1 -\frac{{\beta }_{3}{Q}_{\left(t-1\right)}}{\mathrm{max}\left(Q\right)}\right)+ {\beta }_{4}{T}_{\left(t-1\right)}- {\beta }_{5}{{T}_{\left(t-1\right)}^{2}}+ {\varepsilon }_{l,t}$$

4 $${\varepsilon }_{l} \sim Normal \left(0, {\sigma }_{l,t}\right)$$

Here, ${\beta }_{0}$ is the baseline daily larval survival on the linear scale. A survival probability prior was assigned under zero rainfall and average temperature (i.e. 27 °C) and then calculated the intercept of ${\beta }_{0}$ to reflect this prior information. When there is no effect of any environmental covariates (prior takes values between 0.80 and 1, Table 1). The coefficient ${\beta }_{1}$ quantifies the effect of current rainfall $(R)$*;* with the envisioned scenario being that higher $R$ (i.e. flooding) tends to wash away larvae hence reducing the baseline survival [49]. This ${\beta }_{1}$ was defined by an informative gamma prior with shape = 5.382 and rate of 46.4 (Table 2) which permits anything from no rain effects to 100% mortality. The coefficient ${\beta }_{2}$ quantifies the effect of larval density at time $t$ on larval survival. A monotonic negative relationship was assumed based on the biologically-plausible hypothesis that larval survival is reduced at high larval density because of resource competition and intraspecific cannibalism [29, 50]. This coefficient ${\beta }_{2}$ was defined by an uninformative gamma prior with shape of 0.5 and rate of 1 (Table 2), which allows the impact of density to range from no effect to complete annihilation.

**Table 2:** Priors for the intrinsic and extrinsic drivers of the population dynamic as used in the state-space model of *Anopheles funestus* and the estimated posteriors mean and 95% credible intervals

Parameter		Prior distribution				Posterior distribution	
Notation	Description	Type	Source	Mean	sd	Mean	95-percentiles
${\beta }_{1}$	Linear coefficient for rainfall on larvae survival	Gamma	Uninformative prior	0.1	0.05	0.01681	[0.00604, 0.0308]
${\beta }_{2}$	Density dependent coefficient for larvae on larvae survival	Gamma	Uninformative prior	0.5	0.7	1.0283e−4	[1.0e−4, 1.1205e−4]
${\beta }_{3}$	Coefficient of interaction between larvae and rainfall on larvae survival	Gamma	Uninformative prior	0.9	0.1	0.9601	[0.80810, 0.99999]
${\beta }_{4}$	Linear coefficient for temperature on larvae survival	Gamma	Uninformative prior	1	0.316	0.487	[0.203, 0.806]
${\beta }_{5}$	Quadratic coefficient for temperature on larvae survival		A function of ${\beta }_{4}$	${\beta }_{5}=\frac{{\beta }_{4}}{2*\rho }$		− 0.00902	[− 0.01492, − 0.00376]
${C}_{1}$	Linear coefficient for temperature on larvae development period	Gamma	Uninformative prior	0.001	0.001	5.362e−4	[2.51e−8, 2.311e−3]
${\varphi }_{1}$	Linear coefficient for temperature on unfed, survival	Gamma	Uninformative prior	1	0.316	0.074	[0.068, 0.081]
${\varphi }_{2}$	Quadratic coefficient for temperature on unfed survival	Gamma	A function of ${\varphi }_{1}$	${\varphi }_{2}=\frac{{\varphi }_{1}}{2*\rho }$		− 1.378e−3	[− 1.50e−3, − 1.26e-3]
${\theta }_{1}$	Linear coefficient for temperature on bloodfed survival	Gamma	Uninformative prior	1	0.316	0.074	[0.068, 0.081]
${\theta }_{2}$	Quadratic coefficient for temperature on bloodfed survival	Gamma	A function of ${\theta }_{1}$	${\theta }_{2}=\frac{{ \theta }_{1}}{2*\rho }$		− 1.378e−3	[− 1.50e−3, − 1.26e-3]
${\alpha }_{1}$	Linear coefficient for temperature on gravid survival	Gamma	Uninformative prior	1	0.316	0.074	[0.068, 0.081]
${\alpha }_{2}$	Quadratic coefficient for temperature on gravid survival	Gamma	A function of ${\alpha }_{1}$	${\alpha }_{2}=\frac{{\alpha }_{1}}{2*\rho }$		− 1.378e−3	[− 1.50e−3, − 1.26e−3]

The term inside brackets in Eq. (3) represents the fact that density dependence needs to be modulated by the availability of larval habitat. The availability of suitable aquatic habitats for oviposition will increase with rainfall; thus potentially reducing the crowding of larvae into the remaining habitats that persist during the dry season. This hypothesis has been supported for *An. gambiae s.l*., where their seasonal population dynamics can be explained by models incorporating a rainfall-dependent carrying capacity [25]. Here, the coefficient ${\beta }_{3}$ was a proportion that captures the potential interaction between larval habitat availability (defined as the cumulative rainfall (Q) over the past week) and larval density ($D$). When rain in the recent week has been the maximum observed (i.e.$Q=\mathrm{max}(Q))$, then ($1 -\frac{{\beta }_{3}{Q}_{\left(t-1\right)}}{\mathrm{max}\left(Q\right)}$) would be the smallest amount of density dependency experienced by *An. funestus*. The prior distribution for ${\beta }_{3}$ was defined by an upward-biased beta prior with mean 0.9 and variance of 0.01 allowing ${\beta }_{3}$ to have positive impact on larvae survival.

Additional covariates were incorporated to assess the role of temperature on larval survival (via the coefficients ${\beta }_{4}$,${\beta }_{5}$). The parameter ${\beta }_{4}$ captures the potentially positive effects of temperature on daily larval survival, which were defined by an uninformative gamma prior with mean of 1 and variance of 0.1 considering 27 °C as the optimal temperature ($\rho )$ for maximum survival [51]. This prior allows temperature to vary from having no impact, to high positive impact on larval survival. Alternatively, the relationship between larval survival and temperature may be characterized by survival being reduced at low or very high temperature, and peaking in the middle [52]. The coefficient ${\beta }_{5}$ was incorporated to capture this potential curvilinear relationship but was dependent on ${\beta }_{4}$, to ensure that the optimum temperature was fixed at 27 °C, (${\beta }_{5}=\frac{{\beta }_{4}}{2\rho })$. The prior of ${\beta }_{4}$ allowed extreme temperatures values away from the optimum to range from having no effect to generating 100% mortality. The parameter ${\varepsilon }_{l}$ is capturing the unexplained stochasticity associated with larval survival. This error term was defined by a normal prior with mean of 0 and a precision ${\sigma }_{l}$ from a gamma distribution with both shape and rate of 10.

The linear predictors for survival of unfed, blood-fed, gravid (uppercase ${S}_{a}\left(t\right), {S}_{f}\left(t\right), {S}_{v}(t)$) and daily probabilities of survival (lowercase ${s}_{a}\left(t\right),{ s}_{f}\left(t\right), { s}_{v}\left(t\right)$) were structured similarly to Eq. 3. The daily survival probabilities of adult stages were thus defined as the functions of daily temperature; such that an increase in temperature would result in an increase in the survival of all three adults stages and reduction in survival when temperature become lethal [22, 53]. The biological relationship between adult survival and temperature was assumed to be curvilinear [22, 53, 54].

5 $${S}_{a}\left(t\right)={\varphi }_{0}+{\varphi }_{1}{T}_{\left(t-1\right)} - {\varphi }_{2}{T}_{\left(t-1\right)}^{2}+ {\varepsilon }_{a,t}$$

6 $${S}_{f}\left(t\right)={\theta }_{0}+{\theta }_{1}{T}_{\left(t-1\right)} - {\theta }_{2}{T}_{(t-1)}^{2} + {\varepsilon }_{f,t}$$

7 $${S}_{v}\left(t\right)={\alpha }_{0}+{\alpha }_{1}{T}_{\left(t-1\right)} - {\alpha }_{2}{T}_{\left(t-1\right)}^{2}+ {\varepsilon }_{v,t}$$

8 $${{\varepsilon }^{*}}_{t} \sim Normal \left(0, {\sigma }_{t}^{*}\right)$$

Here, ${\varphi }_{0}, { \theta }_{0},$ and ${\alpha }_{0}$ refer to the baseline survival of unfed, blood-fed and gravid females respectively on a linear scale, under fixed temperature conditions of 27 ± 2 °C (insectary standard under which *An. funestus* have maximum survival [52, 54]), and assumes no blood meal limitation. The positive impact of temperature on all three life stages was represented by the coefficients ${\varphi }_{1},{ \theta }_{1}$ and ${\alpha }_{1}$ with an uninformative gamma prior with mean 12.5 and variance of 6.25. The coefficients ${ \varphi }_{2},{ \theta }_{2}$ and ${\alpha }_{2}$ correspond to the curvilinear effect of temperature on the survival of all three adult stages with their priors derived from the ratio between the linear coefficient and twice optimum temperature. This formulation ensured that the optimum temperature is fixed at (27 °C). The parameters ${\varepsilon }_{a}, { \varepsilon }_{f}, {\varepsilon }_{v}$ capture unexplained variation associated with survival during the distinct gonotrophic stages. These error terms (${\varepsilon }^{*})$ were defined by normal priors with mean of 0 and a precision ${\sigma }^{*}$ from a gamma distribution with both shape and rate of 10 for unfed, blood-fed and gravid females.

#### Development between stages

The daily development probability from one life stage to the next was defined as the reciprocal of the development time (days) between the stages (assuming that all development times take longer than a day). An increase in temperature was assumed to reduce the development period of larvae [47, 53, 55, 56].

9 $$l\left(t\right)=\frac{\mathrm{exp}\left(L\left(t\right)\right)}{1+\mathrm{exp}\left(L\left(t\right)\right)}$$

Specifically, $L\left(t\right)$ is written as the function of temperature covariates:

10 $$L\left(t\right)= {C}_{0}+{C}_{1}{T}_{\left(t-1\right)}$$

Here ${C}_{0}$ corresponds to the baseline daily development period on a linear scale defined by an informative beta prior with range defined in Eq. 16–17 (Table 1). The coefficient ${C}_{1}$ explains the positive effect of temperature on larval development period, with its prior values derived from an uninformative gamma prior with mean 0.001 and standard deviation 0.001.

The development time for other life history stages (eggs and pupae) and the time between gonotrophic stages were assumed to be independent of temperature and other environmental covariates. The numbers of individuals (${\mathcal{K}}_{m}$) graduating from one stage to the next each day were modelled as a binomial process Eq. 11.

11 $${\mathcal{K}}_{m}\left(t\right)\sim Binomial({r}_{m},{ \mathcal{W}}_{m-1}(t)$$

Here the rate ${r}_{m}$ is a development probability as defined in Eq. 11 for $m$ stage, with assigned informative prior values as described through a generic prior in Eq. 16–17 (Table 1). Parameter ${\mathcal{W}}_{m-1}\left(t\right)$ refers to the number surviving the preceding life stage.

### Fecundity

The number of eggs laid at each time step was drawn from a Poisson distribution whose rate was the product of per-capita fecundity (number of eggs laid by blood-fed *An. funestus* under insectary conditions ($${b}_{0}$$), a penalized rate for the egg survival ($${s}_{e})$$, the number of gravid mosquitoes ($${V}_{(t-1)})$$ and ratio of females-males (assumed to be 0.5) as assessed at the pupae stage [19].

12 $${b}_{t}= {\mathrm{exp}(b}_{0}+ {\varepsilon }_{b,t})$$

13 $$B(t)\sim Poisson\left(0.5{b}_{t}{s}_{e}{V}_{(t-1)} \right)$$

The error term ${\varepsilon }_{b}$ was defined by normal prior with mean of 0 and a precision from a gamma distribution with both shape and rate of 10.

### Observation-derived components of the Bayesian SSMs

Observations of the abundance of adults (unfed, bloodfed, gravid) $A$ at time $t$ were modelled as a normal distribution with varying daily means $\mathrm{\overline{a} }$ determined by the biological model and a precision $\tau$ representing observation error. A fixed coefficient of variation ($\xi )$ for the daily observation process was assumed and assigned an uninformative prior with values between 0.1 and 0.9 (Table 1). The CDC light trap typically samples mosquitoes from populations of unknown size, for which the daily catch rates are difficult to quantify independently. A parameter $\vartheta$ was therefore incorporated both into the precision $\tau$ and daily varying means to account for an observed weekly periodicity in adult abundance, which was otherwise hard to interpret. This parameter was allowed to vary both by day of the week $j$ and between the two populations $k$ (2015 and 2018–19 datasets). The $\vartheta$ values were derived from a logit function $\mathrm{ exp}\left(\rho \right)/(1+\mathrm{exp}\left(\rho \right))$, with $\rho$ defined from the uninformative normal prior with mean and standard deviation of 0 and 10 respectively. Therefore precision $\tau$ can be written as $\frac{1}{{({\xi }_{t}{\overline{a} }_{t}{\vartheta }_{jk})}^{2}}$ for all the adult stages. Thereafter, the observation abundance was estimated as follows:

14 $$A(t)\sim Normal\left({\overline{a} }_{t}{\vartheta }_{jk}, \frac{1}{{({\xi }_{t}{\overline{a} }_{t}{\vartheta }_{jk})}^{2}}\right)$$

#### Trap bias

The trapping method (CDC Light traps) primarily targets unfed host-seeking mosquitoes [57]. Blood fed and gravid mosquitoes are assumed to no longer host-seek, and represent a small proportion 0.5–3% of total collections of females caught [6, 22]). To account for these biases in sampling, a new parameter of “trap-biasness”$\omega$ was added in the observation model for both precision $\tau$ and varying daily means ${\overline{a} }_{\eta }$. The prior values for $\omega$ were estimated from independent studies from the same locations [6, 58], and ranged from 0.05–0.15 (Table 1), with variations between the two life stages $\eta$. Therefore, the observation model for blood-fed and gravid (${A}_{\eta })$ was rewritten by modifying Eq. 14 as follows

15 $${A}_{\eta }(t)\sim Normal\left({\overline{a} }_{\eta t}{\vartheta }_{jk}{\omega }_{\eta }, \frac{1}{{({\xi }_{t}{\overline{a} }_{\eta t}{\vartheta }_{jk}{\omega }_{\eta })}^{2}}\right)$$

#### Prior distributions

Since this model contains a large number of parameters, use of un-scaled informative priors restricted model convergence and mixing. A rescaled beta distributions of the informative priors [59] was opted and calibrated as follows:

16 $$Y=Beta \left(5, 5\right)$$

17 $$X={X}_{min}+Y\left({X}_{max}- {X}_{min}\right)$$

where $Y$ is a dummy variable that takes values in the interval [0, 1] with mean of 0.5 and standard deviation of 0.15, selected to provide low likelihood at the values 0 and 1. The values of ${X}_{min}$ and ${X}_{max}$ define the range of the parameter of interest as dictated by the prior information. Since the information on priors was provided in form of mean ($\mu$) and standard deviation ($\sigma$), the values were defined as ${X}_{min},{X}_{max}= \mu \pm 2\sigma$. Survival, development period, trap-biasness, variability in daily catches and fecundity parameters were all assigned priors according to Eq. 17.

### Model selection, model fitting and outputs

Model fitting was done using the R statistical software version 4.0.5 [60]. Population models were fitted using a Markov Chain and Monte Carlo sampling (MCMC) algorithm via the JAGS software [61] interfaced to R via the *runjags* package [62] (code provided in the Additional file 1: Fig. S1). To achieve convergence, the model with 6 chains was run in parallel for ${10}^{5}$ samples with a burn-in of ${10}^{5}$, keeping every 10th iteration for memory-saving reasons. Convergence was assessed by visual investigation of the trace plots, prior-posterior distribution using the *coda* package [63], effective sample sizes and the Gelman Rubin diagnostic [64]. Model comparisons were done using the deviance information criterion (DIC) [65], and the ones with the lowest DIC selected as the most preferred. The predicted and observed densities of *An. funestus* adult females were plotted to evaluate consistent prediction biases visually (Additional file 1: Fig. S2). Posterior means and 95% credible intervals for the key survival parameters, development period, density dependence, environmental covariates (temperature and rainfall) and fecundity were also reported to reveal different dynamical aspects of the system.

## Results

### Population trajectories and seasonal trends

**Fig. 3:**
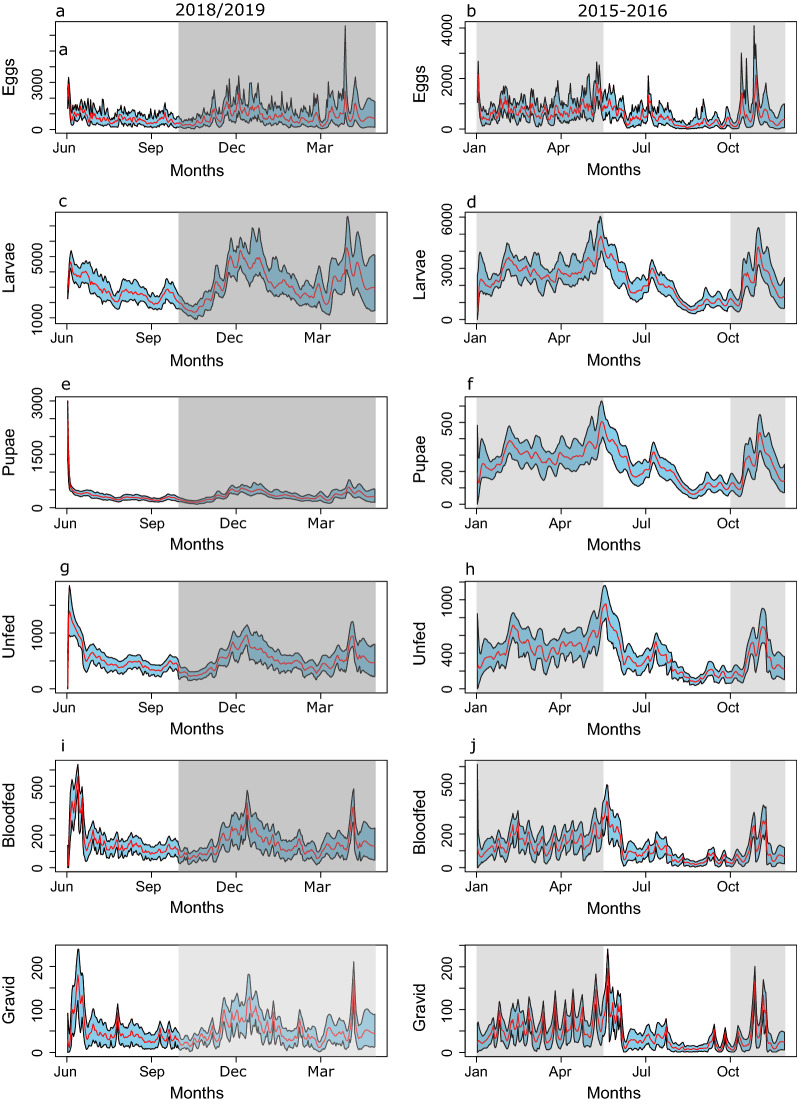
Reconstruction of the abundance trajectories for all the six life-stages. The red line indicates the mean posterior values and the respective 95% confidence intervals are shown in “sky-blue”. Left column (**a**, **c**, **e**, **g**, **i**, **k**) is data collected from June 2018 to May 2019 and right column (**b**, **d**, **f**, **h**, **j**, **l**) is data collected from Jan-Dec 2015. The grey area indicates the period with rainfall

Bayesian state-space model was used to describe the dynamics of wild populations of *An. funestus*. The full results, including summaries of posterior means for all the fitness and demographic parameters are reported in Table 1. The most parsimonious model (model-7, Table 3) included density dependence, and temperature and rainfall (current and lagged) impacts on larval survival, and the effect of temperature on larval development period. The only covariate that was not retained in the “model-7” was temperature impacts on adult survival. This model satisfactorily reconstructed the population dynamics of *An. funestu*s in the study villages, with all environmental covariate relaxation applied based on DIC selection. Population trajectories were estimated for all six *An. funestus* life history and gonotrophic stages after accounting for potential impacts of environmental covariates and density dependence (Fig. 3).

**Table 3:** Model selection: Description of all models fitted with and without environmental covariates and their corresponding delta-Deviance Information Criterion ΔDIC

Model	Removed covariate(s)	Fitness measure	Penalized deviance (pD)/DIC	ΔpD/DIC
Model 1-Full	None		35,500	25,339
Model 2	Temperature	Larval survival	10,555	394
Model 3	Rainfall	Larval survival	10,277	116
Model 4	1 week cumulative rainfall*density dependency	Larval survival	11,886	1725
Model 5	Density dependency	Larval survival	11,011	850
Model 6	Temperature	Larval development period	10,873	712
Model 7^a^	Temperature	Adult survival	10,161	0
Model 8	Model 7—temperature	Larval survival	10,299	138
Model 9	Model 7—rainfall	Larval survival	15,766	5605
Model 10	Model 7—1 week rainfall:density dependency	Larval survival	10,199	38
Model 11	Model 7—density dependency	Larval survival	10,383	222
Model 12	Model 7—Temperature	Larval development	10,497	336

Model 8–12 consists of model-7 minus one more environmental covariate. Model 4 involved the removal of the interaction term

^a^The best model (lowest DIC/Penalized Deviance) value-model-7 followed by model-10, * Interaction between 1 week cumulative rainfall and density dependency

**Fig. 4:**
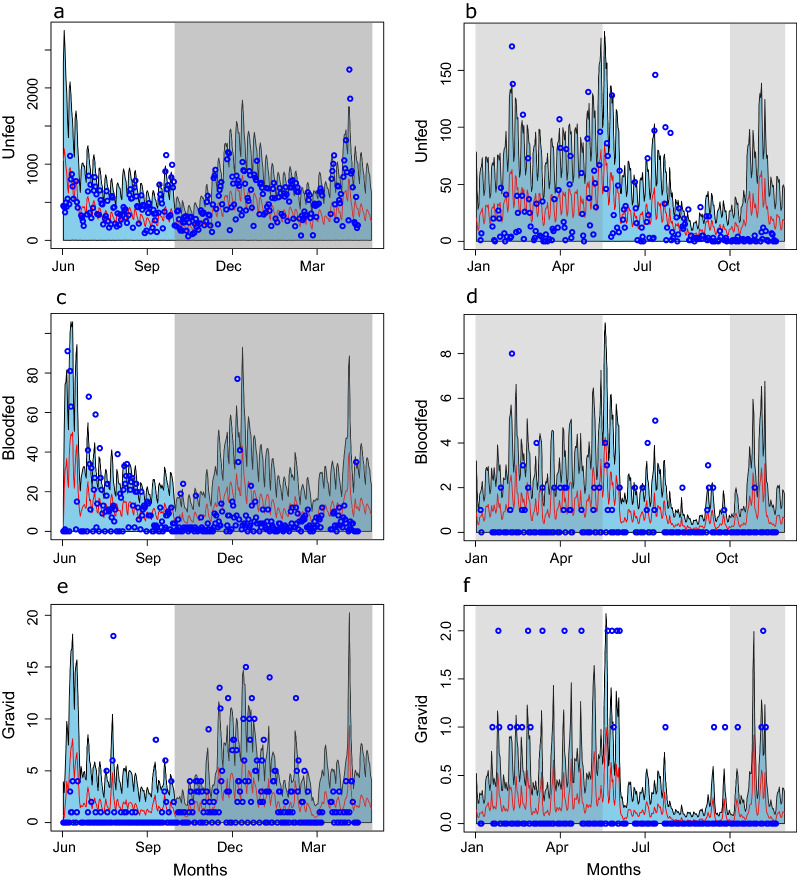
Observed vs. model estimated values for the three adult stages with data collected using CDC light trap both in May 2018 –June 2019 (left column- **a**, **c**, **d**) and Jan-Dec 2015 (right column- **b**, **d**, **e**). Red lines are the model estimated trajectories with “sky-blue” showing their 95% credible intervals. The blue circles are the observed values from the Light trap catches. Grey areas are the periods with rainfalls episodes

These trajectories reflect the annual trend in abundance spanning in periods from low or no rainfall to high rainfall. All trajectories show a relatively high abundance of *An. funestus* right after the rainy season, followed by reduced but sustained abundance during the dry period for all the life stages. After accounting for observation biases during sampling, the observed abundance of unfed, gravid and blood-fed groups largely falls within the credible intervals of the predicted values (Fig. 4).

### Survival and fecundity

**Fig. 5:**
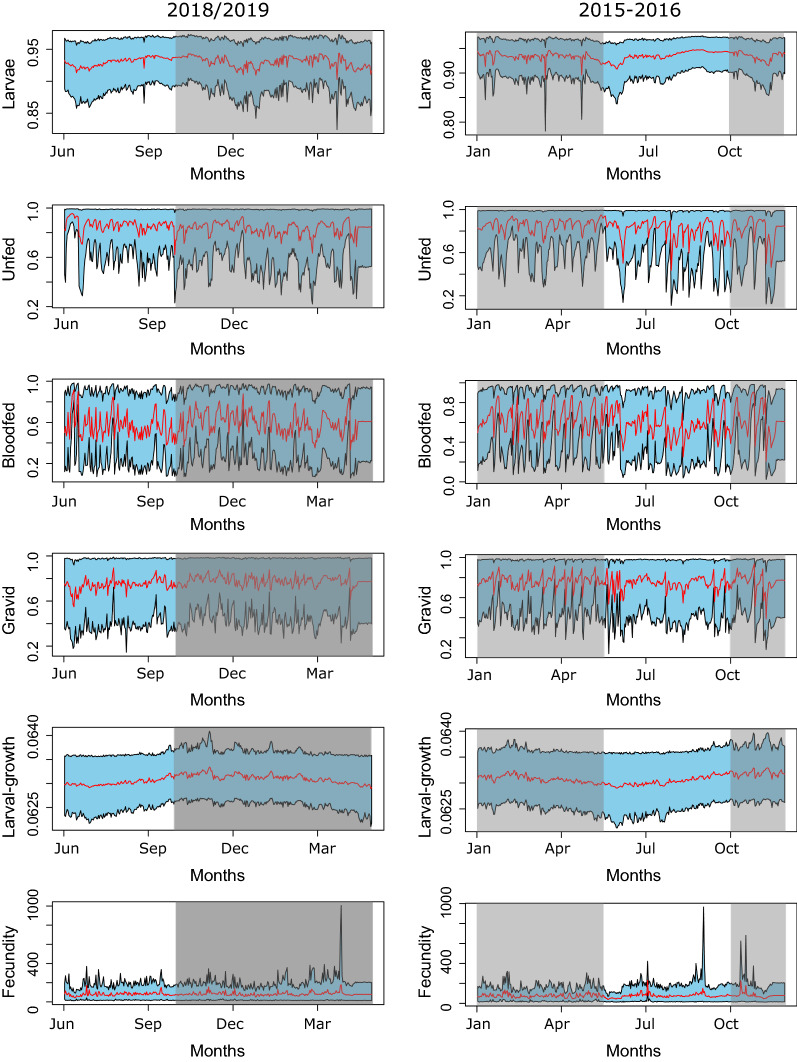
Reconstruction of the survival trajectories for all the four stages (larvae, unfed, bloodfed, and gravid) which were affected by the environmental covariates. The two bottom rows show the larval development period and fecundity trends. Left column (**a**, **c**, **e**, **g**, **i**, **k**) is trajectories from June 2018 to May 2019 and right column (**b**, **d**, **f**, **h**, **j**, **l**) is from Jan-Dec 2015. Grey area is the period with rainfall. Y-axis shows the survival rates of different life stages and the bottom row (**k**, **l**) shows per-capita fecundity

Estimated *An. funestus* larval survival trajectories demonstrate substantial mean variability during the two seasons, with no clear pattern of seasonality (Fig. 5a and b), Table 1). Similarly, the survival trajectories of the adult stages (all gonotrophic states) were variable throughout the year, with daily survival rate ranging from 0.2 to 1.0 and not consistently differing between wet and dry seasons (Fig. 5c to h, Table 1). Per capita fecundity was estimated to be between 75 and 81 eggs per female *An. funestus* (Table 1). While the abundance of this species fluctuated seasonally, per capita fecundity remained consistent throughout the year (Fig. 5k and l).

**Fig. 6:**
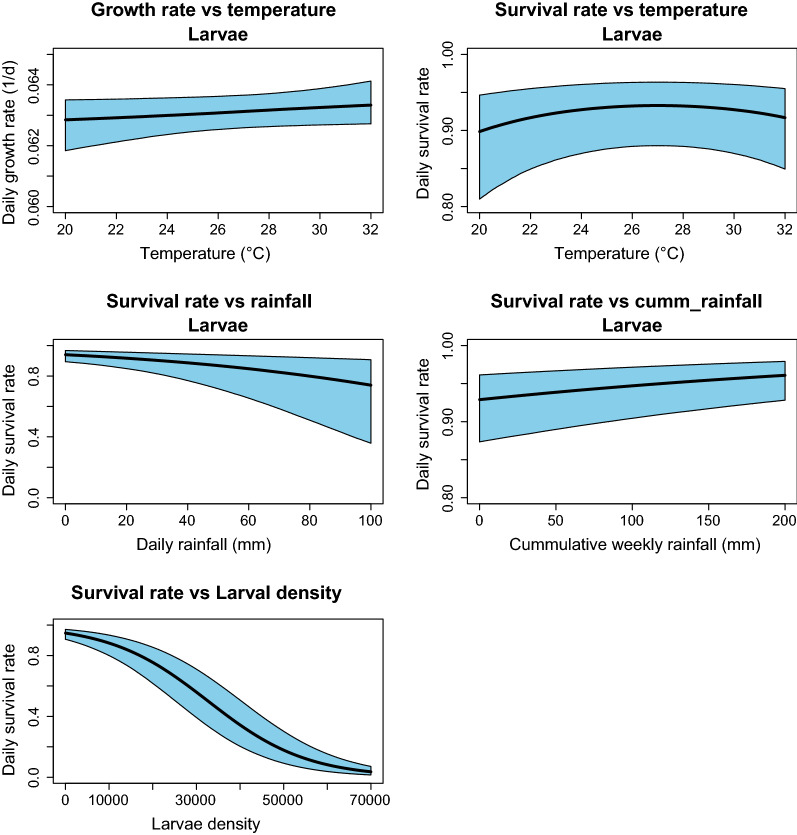
Relationship between environmental covariates and fitness parameters as estimated from the SSMs of population dynamic of *Anopheles funestus*

Temperature was an important predictor of larval survival with a curvilinear relationship (ΔDIC = 138, Table 3, Fig. 6b), and that temperature has a positive monotonic relationship with larval development period (ΔDIC = 336, Table 3, Fig. 6a); with the larval development period estimated to last about 16 days on average. Additionally, daily rainfall was an important driver for the dynamics of *An. funestus* by reducing larval survival (ΔDIC = 5605, Table 3, Fig. 6c) with a negative monotonic relationship.

### Effects of density dependence

Density dependence was the only intrinsic feature incorporated in this dynamic model of *An. funestus*. The model was able to converge efficiently without crashing when density dependency was removed, suggesting this process plays a detectable but relatively minor role population regulation when compared with extrinsic factors (ΔDIC = 222, Table 3, Fig. 6e). To verify this, a simulation was run and discovered that the estimated density dependence was actually quite low, even when simulating with a single dataset. The model fitting process also suggested the interaction parameter (${\beta }_{3})$ between larvae density $(D)$ and one week cumulative rainfall $(Q)$ contributes to *An. funestus* dynamics by positively increasing larval survival (ΔDIC = 38, Table 3, Fig. 6d).

## Discussion

Advanced methods used in mainstream ecological studies was adapted and fitted a state-space model (SSM) to field and laboratory data to accurately reconstruct population dynamics of wild population of *An. funestus.* The SSM inferred the trajectories of multiple life-cycle and gonotrophic stages of wild *An. funestus* females. This allows the reconstruction of the observed trajectories of larvae and adult females for the wild *An. funestus* in Tanzania for the first time. This analysis indicated that the dynamics of *An. funestus* were best explained in a model that included density dependency, temperature (curvilinear relationship) and rainfall (negative monotonic relationship) on larval survival, and temperature on the larval development period (positive monotonic relationship). In contrast, model fit was not improved by incorporating temperature dependency into adult survival (all gonotrophic stages). *Anopheles funestus* abundance vary seasonally between wet and dry but demographics rates (i.e. survivals, fecundity and development period) did not vary after accounting for the impact of environmental covariates and density dependence. These results are very useful for generating hypotheses about the nature and relative magnitude of drivers of *An. funestus* population dynamics in the wild. This model can be extended to include a component on malaria dynamics in humans; or to compare the efficacy and effectiveness of different interventions in combination or singly. This would allow more sophisticated evaluation of suitability of *An. funestus*-specific interventions; including prediction of the potential combined effect of strategies that acting at different life-cycle stages and/or target different demographic processes (e.g. survival versus fecundity).

Extrinsic covariates such as rainfall and temperature were all hypothesised to be the main drivers for the dynamics of this vector species. This study supports the hypothesis that rainfall is a significant driver of the population dynamics of wild *An. funestus*. Overall, the abundance of all life stages were relatively higher in rainy compared to dry periods of the year as previously documented [22, 66–68]. Rainfall covariates were directly included in the larval survival model since it is the only stage on which rainfall was hypothesized to have a significant impact. Daily larval survival as estimated by the SSM showed high variability both within seasons and across the year. There was support for a monotonic association between rainfall and larval survival; characterized as reduction in larval survival during periods of heavy rainfall [69]. Time lags have been used to assess rainfall impacts on the dynamics of these vectors [22]. *Anopheles funestus* abundance have been shown to be positively associated with the cumulative lag rainfall [22]. Here one week cumulative rainfall was included in the model to account for its effect on survival. Similar to other vectors of malaria transmission such as *An. gambiae*, rainfall have always been considered as the main factor regulating the dynamics, despite ecological differences between the two vector species [22, 46, 70, 71].

The SSM also provided support for the hypothesis that temperature is an important driver of *An. funestus* dynamics; although the nature of temperature effects was complex and variable between life history stages. For example, temperature was associated with both larval survival and development, but not adult survival or fitness. Furthermore the estimated impacts of temperature on larval ecology were complex; with the SSM suggesting a curvilinear relationship with survival but a positive monotonic impact with the larval development period. These findings validate the prior studies that demonstrated that temperature had a curvilinear influence on *Anopheles* larval survival, with a rise in temperature above/below the optimum lowering survival [47, 48, 72]. The larval period of *An. gambiae* is temperature dependent [55, 72]; thus this model incorporated a positive monotonic effect such that development is fastest when temperature is high and just below maximum threshold for larval development [72, 73]. In the final model the effect of temperature on gonotrophic stages was not found to be an important driver for the dynamic of this species thus left out during model selection process.

Little is known about the effect of density dependence on *An. funestus* due to its ecology and reliance on the large semi-permanent and permanent breeding habitats [20, 21]. However, density dependence is already well-known to be an important driver for dynamic of other malaria vectors like *An. gambiae* [25, 27–29, 31, 67, 74] and other non-malaria vectors like *Aedes aegypti* [75, 76]. Variations in densities during the aquatic stages of *An. gambiae s.l.* have been found to affect adult fitness [28, 77]. The SSM fit better when density dependence of larval survival was included, though the relative magnitude of this process was quite small and likely to have minor impact on the overall dynamics of *An. funestus* populations (Fig. 6e). These findings suggest that *An. funestus* populations are likely to be regulated more by extrinsic than intrinsic processes. These findings corroborate the original hypotheses about density dependence having a weaker regulatory role in this species on the basis of the types of larval habitats (i.e. larger and more permanent habitat [20, 78]), which can likely sustain higher resources and thus reduce competition than in *An. gambiae*. This is the first report documenting the role of density dependence on the dynamics of the wild populations of *An. funestus*. Now that colonies are becoming more feasible, more thorough investigation on the role of density dependence in the dynamic of *An. funestus* is prerequisite.

In addition to highlighting potential drivers of *An. funestus* populations, the SSM here generated plausible estimates of key demographic and life-history process in the wild. This model estimated that *An. funestus* larvae takes an average of 15.6–16.1 days to grow from first instar larvae to pupae; which is relatively long compared to the other major vectors in the *An. gambiae* complex (9–11 days [48, 55]). This apparently longer development period of *An. funestus* may be a product of their adaptation to more permanent, year-round breeding habitats that are unlikely to dry up; thus reducing selection for rapid development. The SSM estimated that the daily survival of wild *An. funestus* larvae could be as high as 0.95, compared to the 0.83 [0.80, 0.86] mean daily survival rate of the known vector of malaria transmission *An. gambiae* [48, 79]. This matches observations from insectary experiments in which *An. funestus* larvae have higher survival than *An. gambiae* [19, 38, 80]. Given the apparently higher rates of survival in *An. funestus* than in *An. gambiae*, these findings suggest that more lethal intervention may be required to control *An. funestus* both at larvae and adults stages.

The impact of any vector control largely depends on the ecology of the specific vector species. Differences in ecology between *An. gambiae* and *An. funestus* are likely to affect the relative impact of interventions. For instance, *An. gambiae* prefer breeding in small and temporary habitats which dry up quickly when there is no rainfall which is opposite to *An. funestus* habitats. Despite the fact that *An. funestus* habitats are "few, fixed, and findable" and might be easily targeted for larviciding [20] during the dry season, treating habitats such as rivers or bigger ponds could pose logistical challenges. The persistence of *An. funestus* throughout the year even during the driest periods suggest this vector is less seasonal compared to *An. gambiae s.l*., which experience much more dramatic “boom and bust” dynamics in relation to seasonal rains [6, 22, 68]. If interpreted together with the observation that survival estimates were not seasonal, the model suggests that this species is likely responsible for year-round malaria transmission throughout the year, while other species, which mostly occupy temporary habitats may be responsible for any apparent seasonality in transmission.

Models of vector population dynamics can provide a useful guide for the selection of optimal vector control strategies; particular through enabling more focal investigation of the benefits of seasonal or spatial targeting and use of combined versus single interventions. Despite its complexity, this population dynamics model provides a useful framework for investigation of the stability of *An. funestus* populations. With additional data, this model can be further refined to include additional modifications related to vector ecology and behaviour that may impact intervention (e.g. host choice and its impacts on fitness, predation during larval or adult phase and spatial components). Such further elucidation may increase the predictive accuracy of this SSM in specific contexts, but even the more general framework developed here have flexibility to introduce stage-specific mortality effects expected from different types of vector control [16, 32, 33]. For example, this framework could be used to model the impact of combined interventions including those that target adult females (insecticide-treated nets (ITNs), IRS) and larviciding; and assessment of how mortality varies with different coverage [16, 32]. It can also be used to investigate the possible response of vector population to climate change anticipated in Tanzania and other African countries. An important limitation of this study is lack of knowledge on what percentage of *An. funestus* mosquito population is sampled by the trap, which is important for understanding the relative magnitude of demographic stochasticity in modelled dynamics. This highlights the need to explicitly incorporate this source of uncertainty into vector and transmission dynamics; including the need for further calibration and standardization of the efficiency and biases associated with particular mosquito trapping methods.

## Conclusions

This study used Bayesian State Space Models (SSM) parameterized with empirical data to quantify key demographic and fitness processes underpinning the population dynamics of *An. funestus* in Tanzania. This is the first use of SSM to understand the population dynamic of the wild vector of residual malaria transmission, *An. funestus* in Tanzania. The model structure allowed investigation of the relative importance of seasonally-varying environmental covariates (i.e. rainfall and temperature) and density dependence; providing some support for both processes although the magnitude of the former was much greater than the latter. The ability of this model to accurately reconstruct the seasonal dynamics and demography of *An. funestus* indicate its value for simulating the response of these populations to vectors control measures applied either individually or in combination. Additionally, the relatively limited evidence of seasonality in key fitness and demographic rates further corroborate evidence that this vector species can facilitate efficient year-round transmission of malaria. Finally, this model also highlights the clear importance of accounting for regional and daily observation biases when modelling mosquito population dynamics.

## Supplementary Information

**Additional file 1: Figure S1.** SSMs model development R codes. **Figure S2.** Goodness-of-fit: Observed versus predicted unfed, bloodfed and gravid densities across all populations. Adjusted R-squared, intercept and slope values are from a linear model of the predicted against observed values. Dotted lines correspond to 1:1 line. Left column (a,c,d) is data collected from June 2018 to May 2019 and right column (b,d,e) is data from Jan-Dec 2015. Grey area is the period with rainfall. **Figure S3.1.** Prior (orange histogram) and posterior (blue histogram) distribution of the main baseline and observational parameters in the state-space model. **Figure S3.2.** Prior (orange histogram) and posterior (blue histogram) distribution of the main environmental covariates parameters in the state-space model.

## Data Availability

The R-codes used for model development are freely available in the supplementary file. The time series data is available upon reasonable request to the author.
