# Supplementary material for: Using Bayesian state-space models to understand the population dynamics of the dominant malaria vector, Anopheles funestus in rural Tanzania
Source: Malar J. 2022 Jun 3;21:161. doi: 10.1186/s12936-022-04189-4 (PMC9166306; doi:10.1186/s12936-022-04189-4)
Supplement: Supplementary file 1 — Additional file 1: Figure S1. SSMs model development R codes. Figure S2. Goodness-of-fit: Observed versus predicted unfed, bloodfed and gravid densities across all populations. Adjusted R-squared, intercept and slope values are from a linear model of the predicted against observed values. Dotted lines correspond to 1:1 line. Left column (a,c,d) is data collected from June 2018 to May 2019 and right column (b,d,e) is data from Jan-Dec 2015. Grey area is the period with rainfall. Figure S3.1. Prior (orange histogram) and posterior (blue histogram) distribution of the main baseline and observational parameters in the state-space model. Figure S3.2. Prior (orange histogram) and posterior (blue histogram) distribution of the main environmental covariates parameters in the state-space model. [file 12936_2022_4189_MOESM1_ESM.pdf]

Supplementary information

Using Bayesian state-space models to understand the population dynamics of the dominant malaria vector, *Anopheles funestus* in rural Tanzania

Halfan S. Ngowo[1,2*], Fredros O. Okumu[1,2,3,4], Emmanuel E. Hape[1,2], Issa H. Mshani[1,2], Heather M. Ferguson[1,2], Jason Matthiopoulos[2]

* Corresponding author

Affiliations

1. Department of Environmental Health & Ecological Sciences, Ifakara Health Institute, Tanzania
2. Institute of Biodiversity, Animal Health and Comparative Medicine, University of Glasgow, United Kingdom
3. School of Public Health, University of the Witwatersrand, Republic of South Africa
4. School of Life Science and Bioengineering, Nelson Mandela African Institution of Science & Technology, Tanzania

Email address:

1. Halfan Ngowo: hngowo@ihi.or.tz
2. Fredros Okumu: fredros@ihi.or.tz
3. Emmanuel Hape: ehape@ihi.or.tz
4. Issa Mshani: imshani@ihi.or.tz
5. Heather Ferguson: Heather.Ferguson@glasgow.ac.uk
6. Jason Matthiopoulos: Jason.Matthiopoulos@glasgow.ac.uk

S1: JAGS code

```
########## 1. Jags model############

popMod2<-"model{
   #Multiple trajectory

   for(i in 1:Ntr){
     ###MAIN LOOP###

      #Supply the initial values
      Ieggs[i,1]~dunif(0,3000)
      Ilarvae[i,1]~dunif(0,3000)
      Ipupae[i,1]~dunif(0,3000)
      Iadult[i,1]~dunif(0,1000)
      Iblood_fed[i,1]~dunif(0,1000)
      Igravid[i,1]~ dunif(0,100)
      IgraG[i,1]~ dunif(0,100)

      eggs[i,1]<-round(Ieggs[i,1])
      larvae[i,1]<-round(Ilarvae[i,1])
      pupae[i,1]<-round(Ipupae[i,1])
      adult[i,1]<-round(Iadult[i,1])
      blood_fed[i,1]<-round(Iblood_fed[i,1])
      gravid[i,1]<-round(Igravid[i,1])
      graG[i,1]<-round(IgraG[i,1])

      ####Start of the time loop#####
      for(t in 2:(tmax))
      {

      larE[i,t]~dnorm(0,precLE+1)
      pupE[i,t]~dnorm(0,precPE+1)
      aduE[i,t]~dnorm(0,precAE+1)
      bloE[i,t]~dnorm(0,precAE+1)
      graE[i,t]~dnorm(0,precAE+1)
      breE[i,t]~dnorm(0,precBE+1)

      #Effect of density dependence, rainfall and temperature on larvae survival
      logit(spv[i,t])<-slInv + PrTempls*Temp[i,t-1] + PrTemps*Temp[i, t-1]^2 - Pr
_dd*prec[i,t-1] - larvae[i,t-1]*Ldd*(1 - Pr_dd3*prec3[i,t-1]/max(prec3))+larE[i,
t]

      larS[i,t] ~ dbin(spv[i,t], larvae[i,t-1])

      logit(spu[i,t])<-sp0+pupE[i,t]
      pupS[i,t] ~ dbin(spu[i,t], pupae[i,t-1])

      #seasonality into adult-unfed
      logit(sav[i,t])<-saInv + 0*Temp[i,t-1] + 0*Temp[i, t-1]^2+aduE[i,t]
      aduS[i,t] ~ dbin(sav[i,t], adult[i,t-1])
```

```
    #seasonality into adult-bloodfed
    logit(sbv[i,t])<-sbInv + 0*Temp[i,t-1] + 0*Temp[i, t-1]^2+bloE[i,t]
    bloS[i,t] ~ dbin(sbv[i,t], blood_fed[i,t-1])

    #seasonality into adult-gravid
    logit(sgv[i,t])<-sgInv + 0*Temp[i,t-1] + 0*Temp[i, t-1]^2+graE[i,t]
    graS[i,t] ~ dbin(sgv[i,t], gravid[i,t-1])

    #Growth for each stage
    eggG[i,t] ~ dbin(lambda, eggs[i,t-1])

    #Positive effect of temp on larval growth
    logit(lgv[i,t]) <- lrInv + PrTemplg*(Temp[i,t-1]-27)
    larG[i,t] ~ dbin(lgv[i,t], larS[i,t])

    pupG[i,t] ~ dbin(pr, pupS[i,t])

    aduG[i,t] ~ dbin(ar, aduS[i,t])

    bloG[i,t] ~ dbin(bfr, bloS[i,t])

    graG[i,t] ~ dbin(gr, graS[i,t])

    #fecundity penalised by egg survival
    log(bre[i,t])<-bre0+breE[i,t]
    fec[i,t] ~ dpois(se*0.5*bre[i,t]*graG[i,t-1])

    # Propagation of population classes through time (no class is allowed to go
extinct, hence the +1)
    eggs[i,t] <-eggs[i,t-1]-eggG[i,t]+fec[i,t]+1
    larvae[i,t] <- larS[i,t]-larG[i,t]+eggG[i,t]+1
    pupae[i,t] <- pupS[i,t]-pupG[i,t]+larG[i,t]+1
    adult[i,t] <- aduS[i,t]-aduG[i,t]+pupG[i,t] + graG[i,t]+1
    blood_fed[i,t] <- bloS[i,t]-bloG[i,t]+aduG[i,t]+1
    gravid[i,t] <- graS[i,t]-graG[i,t]+bloG[i,t]+1

    #Observation process model
    # Observaton likelihoods against real data
    adultD[i,t] ~ dnorm(ef0[i,efLev[t]]*adult[i,t], 1/(seAd[i,t])^2)T(0,)
    blood_fedD[i,t] ~ dnorm(ef0[i,efLev[t]]*blood_fed[i,t]*ef1, 1/(seBf[i,t])^2
)T(0,)
    gravidD[i,t] ~ dnorm(ef0[i,efLev[t]]*gravid[i,t]*ef2, 1/(seGr[i,t])^2)T(0,)

    # Predictions (no data) used to generate prediction intervals for plotting
later
    adultd[i,t] ~ dnorm(ef0[i,efLev[t]]*adult[i,t], 1/(seAd[i,t])^2)T(0,)
    blood_fedd[i,t] ~ dnorm(ef0[i,efLev[t]]*blood_fed[i,t]*ef1, 1/(seBf[i,t])^2
)T(0,)
```

```
gravidd[i,t] ~ dnorm(ef0[i,efLev[t]]*gravid[i,t]*ef2, 1/(seGr[i,t])^2)T(0,)

# Modelling the standard errors for the weekly mean counts.
seAd[i,t]<-1e-6+ef0[i,efLev[t]]*adult[i,t]*cva[i]
seBf[i,t]<-1e-6+ef0[i,efLev[t]]*blood_fed[i,t]*ef1*cva[i]
seGr[i,t]<-1e-6+ef0[i,efLev[t]]*gravid[i,t]*ef2*cva[i]

} #End of time loop


} #End of trajectory loop

 #Density dependence at larval stage (Determines carrying capacity)
Ldd ~ dgamma(0.5,1)T(0.0001,10)


# Precisions for stochasticity in survival rates
precLE~dgamma(10,1)
precPE~dgamma(10,1)
precAE~dgamma(10,1)
precBE~dgamma(10,1)

# Scaling parameters for all beta priors
b1<-100
b2<-100
b3<-2
b4<-2


#Prior for coefficient of variation in trapping
mu.cva<-0.5
sd.cva<-0.4/b3
cvamax<-mu.cva+b4*sd.cva
cvamin<-max(0, mu.cva-b4*sd.cva)
cvae[1]~dbeta(b1, b2)
cva[1]<-cvamin+cvae[1]*(cvamax-cvamin)
cvae[2]~dbeta(b1, b2)
cva[2]<-cvamin+cvae[2]*(cvamax-cvamin)

ef0[1,1]<-0.5

dev[2,1]~dnorm(0,10)
ef0[2,1]<-0.1*exp(dev[2,1])/(1+exp(dev[2,1]))

for( day in 2:7)
{
  dev[1,day]~dnorm(0,10)
  logit(ef0[1,day])<-dev[1,day]
  dev[2,day]~dnorm(0,10)
```

```
    ef0[2,day]<-0.1*exp(dev[2,day])/(1+exp(dev[2,day]))
  }


  # CDC trap effectiveness for blood fed
  mu.ef1<-0.1
  sd.ef1<-0.05/b3
  efmax1<-min(1, mu.ef1+b4*sd.ef1)
  efmin1<-max(0, mu.ef1-b4*sd.ef1)
  efe1~dbeta(b1, b2)
  ef1<-efmin1+efe1*(efmax1-efmin1)

  ef2<-0.505*ef1

  #Proportion eggs survived (literature)
  mu.se<- 0.7940
  sd.se<- 0.101/b3
  semax<-min(1, mu.se+b4*sd.se)
  semin<-max(0, mu.se-b4*sd.se)
  see~dbeta(b1, b2)
  se<-semin+see*(semax-semin)

  #Hatching period
  mu.lam<- 0.5
  sd.lam<- 0.1/b3
  lammax<-min(1, mu.lam+b4*sd.lam)
  lammin<-max(0, mu.lam-b4*sd.lam)
  lamm~dbeta(b1, b2)
  lambda<-lammin+lamm*(lammax-lammin)

  #Surival rates pupae
  mu.sp<-0.9414
  sd.sp<-0.0339/b3
  spmax<-min(1, mu.sp+b4*sd.sp)  #spmax goes to 1.0092
  spmin<-max(0, mu.sp-b4*sd.sp)
  spp~dbeta(b1, b2)
  sp<-spmin+spp*(spmax-spmin)
  sp0<-log(sp/(1-sp))

  #Survival rates adults/unfed
  mu.sa<-0.9348
  sd.sa<-0.0288/b3
  samax<-min(1, mu.sa+b4*sd.sa)
  samin<-max(0, mu.sa-b4*sd.sa)
  saa~dbeta(b1, b2)
  sa<-samin+saa*(samax-samin)
  saInv0<-log(sa/(1-sa))

  #Survival rates blood-fed
```

```
mu.sb<-0.8074
sd.sb<-0.0766/b3
sbmax<-min(1, mu.sb+b4*sd.sb)
sbmin<-max(0, mu.sb-b4*sd.sb)
sbb~dbeta(b1, b2)
sb<-sbmin+sbb*(sbmax-sbmin)
sbInv0<-log(sb/(1-sb))

#Survival rates gravid
mu.sg<-0.9041
sd.sg<-0.0282/b3
sgmax<-min(1, mu.sg+b4*sd.sg)
sgmin<-max(0, mu.sg-b4*sd.sg)
sgg~dbeta(b1, b2)
sg<-sgmin+sgg*(sgmax-sgmin)
sgInv0 <-log(sg/(1-sg))

#Seasonality in adult survival (Temperature)
ATopt<-27 # Optimal temperature for adult survival
PrTempa~dgamma(25,0.5) # Linear term coefficient
PrTempas<- -PrTempa/(2*ATopt) # Quadratic term coefficient
saInv <-saInv0-PrTempa*ATopt/2 # Ensures that the maximum survival is mu.sa
sbInv <-sbInv0-PrTempa*ATopt/2 # Ensures that the maximum survival is mu.sb
sgInv <-sgInv0-PrTempa*ATopt/2 # Ensures that the maximum survival is mu.sg

#Survial rates larvae
mu.sl<-0.9226
sd.sl<-0.0610/b3
slmax<-min(1, mu.sl+b4*sd.sl)
slmin<-max(0, mu.sl-b4*sd.sl)
sll~dbeta(b1, b2)
sl<-slmin+sll*(slmax-slmin)
slInv0<-log(sl/(1-sl))

#Seasonality in larvae (Temperature)
LTopt<-27 # Optimal temperature
PrTempls~dgamma(10,10) # Linear term coefficient
PrTemps<- -PrTempls/(2*LTopt) # Quadratic term coefficient
slInv<-slInv0-PrTempls*LTopt/2 # Ensures that the maximum survival is mu.sl

#Growth rates larvae
mu.lr<-0.0629
sd.lr<-0.0038/b3
lrmax<-min(1, mu.lr+b4*sd.lr)
lrmin<-max(0, mu.lr-b4*sd.lr)
lrr~dbeta(b1, b2)
lr<-lrmin+lrr*(lrmax-lrmin)
lrInv<-log(lr/(1-lr))
```

```
# Temperature dependence of larval growth rate
PrTemplg~dgamma(1,1000) #

#Growth rates pupae
mu.pr<-0.5222
sd.pr<-0.1347/b3
prmax<-min(1, mu.pr+b4*sd.pr)
prmin<-max(0, mu.pr-b4*sd.pr)
prr~dbeta(b1, b2)
pr<-prmin+prr*(prmax-prmin)

#Growth rates adult
mu.ar<- 0.2
sd.ar<-0.01/b3
armax<- min(1, mu.ar+b4*sd.ar)  #1
armin<- max(0, mu.ar-b4*sd.ar)  #6 days=0.1667
arr~dbeta(b1, b2)
ar<-armin+arr*(armax-armin)

#Growth rates blood-fed
mu.bfr<-0.25
sd.bfr<-0.1/b3
bfrmax<-min(1, mu.bfr+b4*sd.bfr)
bfrmin<-max(0, mu.bfr-b4*sd.bfr)
bfrr~dbeta(b1, b2)
bfr<-bfrmin+bfrr*(bfrmax-bfrmin)

#Growth rates gravid
mu.gr<-0.3333
sd.gr<-0.1/b3
grmax<-min(1, mu.gr+b4*sd.gr)
grmin<-max(0, mu.gr-b4*sd.gr)
grr~dbeta(b1, b2)
gr<-grmin+grr*(grmax-grmin)

#Number of eggs
mu.b<-80
sd.b<-20/b3
bmax<-mu.b+b4*sd.b
bmin<-max(0, mu.b-b4*sd.b)
br~dbeta(b1, b2)
breed<-bmin+br*(bmax-bmin)
bre0<-log(breed)

#Effect of precipitation on larvae
Pr_dd~dgamma(5.382, 46.4)

#Density dependence of larvae at lag time 3 days rainfall
Pr_dd3~dbeta(7.2,0.8) # Upward-biased prior, towards 1
```

```
     #data# blood_fedD, gravidD, adultD, prec, prec3, Temp, tmax, Ntr, efLev

     #monitor# breed, se, sp, sa, sb, sg, sl, lr, pr, ar, bfr, gr,lambda, Pr_dd,
Pr_dd3, Ldd, ef1, ef0, precLE, precAE,precBE,precPE
     #monitor# PrTempls, PrTemps, PrTemplg, PrTempa, PrTempas,cva
     #monitor# eggs, larvae, pupae, adult, blood_fed, gravid
     #monitor# spv, sgv, sav, sbv, lgv, lrInv, saInv, sgInv, slInv, sbInv, bre,
bre0
     #monitor# adultd, blood_fedd, gravidd

     #inits# Ldd

     }" #End of the model loop ,

 #Data
 adultD<-rbind(po$adult, p1$adult)
 blood_fedD<-rbind(po$blood_fed, p1$blood_fed)
 gravidD<-rbind(po$gravid, p1$gravid)
 prec<-rbind(po$Rainfall, p1$Rainfall)
 prec3<-rbind(po$RainCuWeek, p1$RainCuWeek)

 tmax<-365
 Ntr<-2

 # Weekly index
 efLev<-c(rep(1:7, 52),1)

 #Temperature
 Temp<-rbind(po$Temp, p1$Temp)

 #standard error
 seAd<-rbind(po$seAd, p1$seAd)/1
 seBf<-rbind(po$seBf, p1$seBf)/1
 seGr<-rbind(po$seGr, p1$seGr)/1

 ############   2. Model running   ############
 n.chains=6
 Ldd<-list(chain1=c(0.01), chain2=c(0.03),
           chain3=c(0.5),chain4=c(0.05),
          chain5=c(0.01),chain6=c(0.02))

 brn=100000  # Burn-in
 spl=10000  # Sampling

 results <- run.jags(popMod2, n.chains=n.chains, adapt = 1000,
                     burnin=brn, sample=spl, thin=10,
                     method = "parallel")
```

Figure S2: Goodness-of-fit: Observed versus predicted unfed, bloodfed and gravid densities across all populations. Adjusted R-squared, intercept and slope values are from a linear model of the predicted against observed values. Dotted lines correspond to 1:1 line. Left column (a,c,d) is data collected from June 2018 to May 2019 and right column (b,d,e) is data from Jan-Dec 2015. Grey area is the period with rainfall.

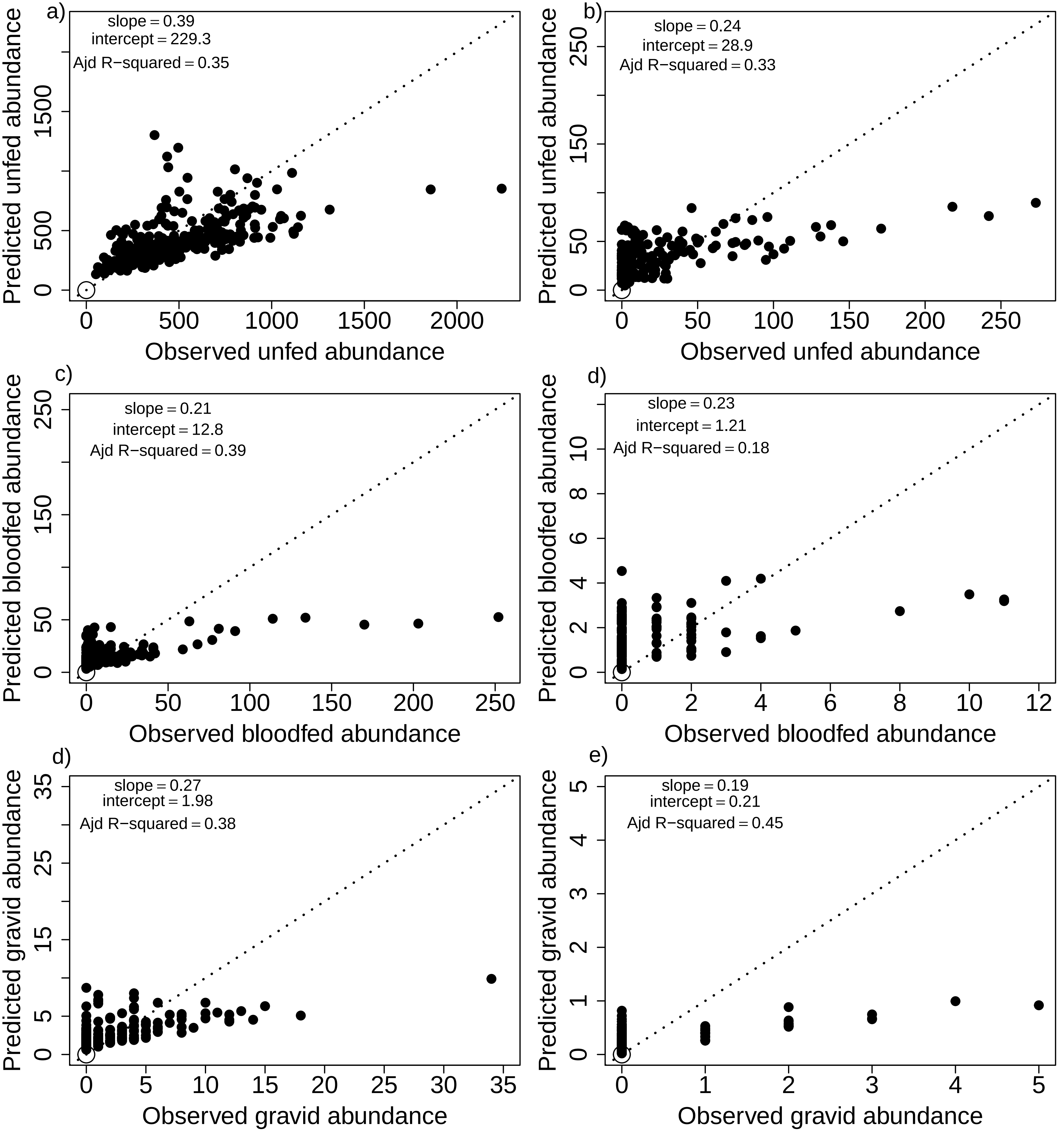

a)
slope = 0.39
intercept = 229.3
Ajd R−squared = 0.35
Predicted unfed abundance
Observed unfed abundance
0
500
1000
1500
2000
b)
slope = 0.24
intercept = 28.9
Ajd R−squared = 0.33
Predicted unfed abundance
Observed unfed abundance
0
50
100
150
200
250
c)
slope = 0.21
intercept = 12.8
Ajd R−squared = 0.39
Predicted bloodfed abundance
Observed bloodfed abundance
0
50
100
150
200
250
d)
slope = 0.23
intercept = 1.21
Ajd R−squared = 0.18
Predicted bloodfed abundance
Observed bloodfed abundance
0
2
4
6
8
10
12
d)
slope = 0.27
intercept = 1.98
Ajd R−squared = 0.38
Predicted gravid abundance
Observed gravid abundance
0
5
10
15
20
25
30
35
e)
slope = 0.19
intercept = 0.21
Ajd R−squared = 0.45
Predicted gravid abundance
Observed gravid abundance
0
1
2
3
4
5

Figure S3.1: Prior (orange histogram) and posterior (blue histogram) distribution of the main baseline and observational parameters in the state-space model.

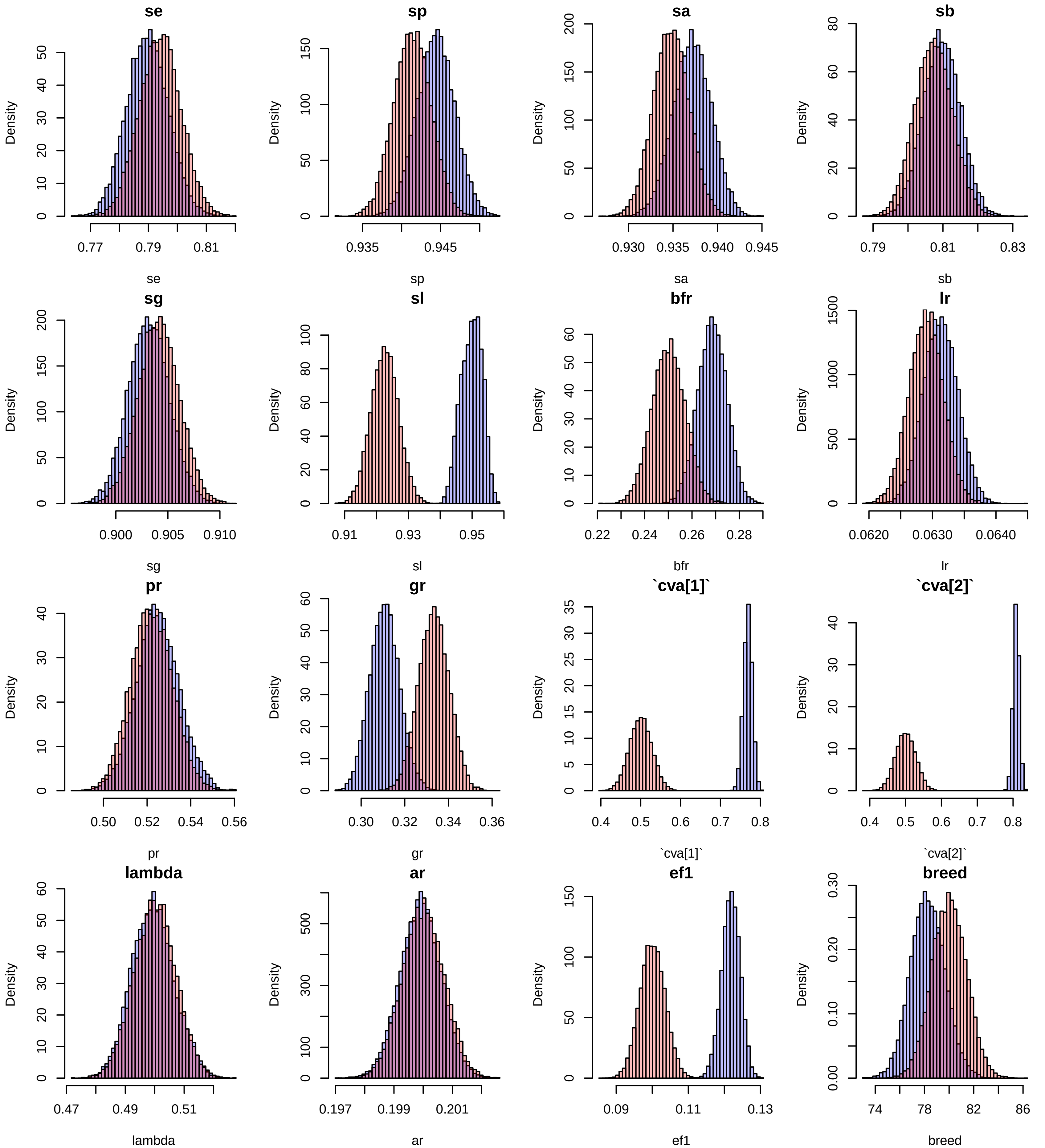
se
Density
0 10 20 30 40 50
0.77 0.79 0.81
se
sp
Density
0 50 100 150
0.935 0.945
sp
sa
Density
0 50 100 150 200
0.930 0.935 0.940 0.945
sa
sb
Density
0 20 40 60 80
0.79 0.81 0.83
sb
sg
Density
0 50 100 150 200
0.900 0.905 0.910
sg
sl
Density
0 20 40 60 80 100
0.91 0.93 0.95
sl
bfr
Density
0 10 20 30 40 50 60
0.22 0.24 0.26 0.28
bfr
lr
Density
0 500 1000 1500
0.0620 0.0630 0.0640
lr
pr
Density
0 10 20 30 40
0.50 0.52 0.54 0.56
pr
gr
Density
0 10 20 30 40 50 60
0.30 0.32 0.34 0.36
gr
`cva[1]`
Density
0 5 10 15 20 25 30 35
0.4 0.5 0.6 0.7 0.8
`cva[1]`
`cva[2]`
Density
0 10 20 30 40
0.4 0.5 0.6 0.7 0.8
`cva[2]`
lambda
Density
0 10 20 30 40 50 60
0.47 0.49 0.51
lambda
ar
Density
0 100 300 500
0.197 0.199 0.201
ar
ef1
Density
0 50 100 150
0.09 0.11 0.13
ef1
breed
Density
0.00 0.10 0.20 0.30
74 78 82 86
breed

Figure S3.2: Prior (orange histogram) and posterior (blue histogram) distribution of the main environmental covariates parameters in the state-space model.

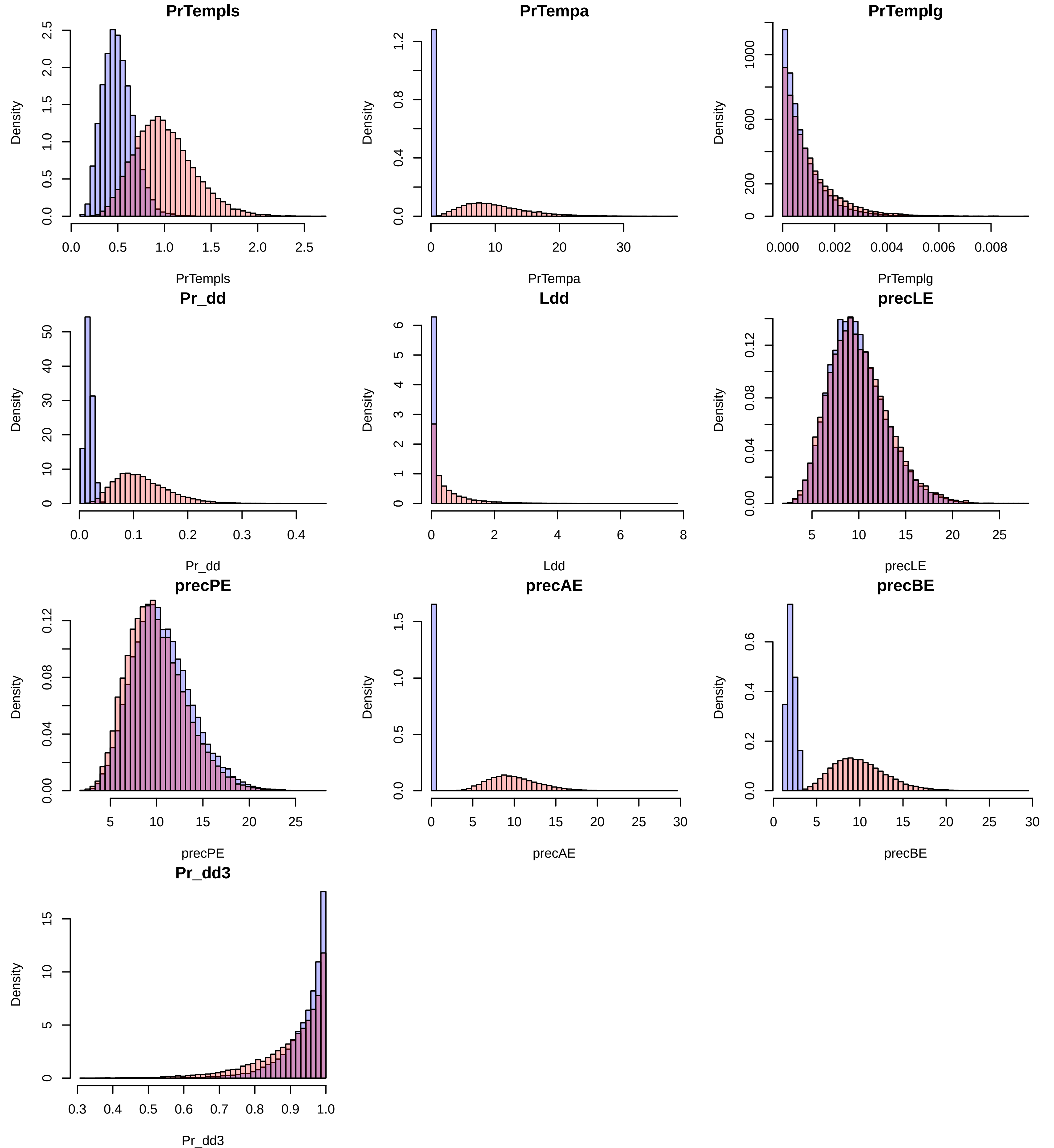
PrTempls
PrTempa
PrTemplg
Pr_dd
Ldd
precLE
precPE
precAE
precBE
Pr_dd3
Density
